# Dopamine Autoxidation Is Controlled by Acidic pH

**DOI:** 10.3389/fnmol.2018.00467

**Published:** 2018-12-18

**Authors:** Nejc Umek, Blaž Geršak, Neli Vintar, Maja Šoštarič, Janez Mavri

**Affiliations:** ^1^Department of Anesthesiology and Surgical Intensive Therapy, University Medical Centre Ljubljana, Ljubljana, Slovenia; ^2^Institute of Anatomy, Faculty of Medicine, University of Ljubljana, Ljubljana, Slovenia; ^3^Department of Anesthesiology and Reanimatology, Faculty of Medicine, University of Ljubljana, Ljubljana, Slovenia; ^4^National Institute of Chemistry, Ljubljana, Slovenia

**Keywords:** dopamine, aminochrome, neurodegeneration, pH, Parkinson disease, reactive oxygen species, oxidative stress

## Abstract

We studied the reaction mechanism of dopamine autoxidation using quantum chemical methods. Unlike other biogenic amines important in the central nervous system, dopamine and noradrenaline are capable of undergoing a non-enzymatic autoxidative reaction giving rise to a superoxide anion that further decomposes to reactive oxygen species. The reaction in question, which takes place in an aqueous solution, is as such not limited to the mitochondrial membrane where scavenging enzymes such as catalase and superoxide dismutase are located. With the experimental rate constant of 0.147 s^−1^, the dopamine autoxidation reaction is comparably as fast as the monoamine oxidase B catalyzed dopamine decomposition with a rate constant of 1 s^−1^. By using quantum chemical calculations, we demonstrated that the rate-limiting step is the formation of a hydroxide ion from a water molecule, which attacks the amino group that enters intramolecular Michael addition, giving rise to a pharmacologically inert aminochrome. We have shown that for dopamine stability on a time scale of days, it is essential that the pH value of the synaptic vesicle interior is acidic. The pathophysiologic correlates of the results are discussed in the context of Parkinson's disease as well as the pathology caused by long-term amphetamine and cocaine administration.

## Introduction

Dopaminergic neurons, that project to many central nervous system (CNS) areas, have been implicated in a myriad of neurologic diseases including Parkinson's disease, dementia with Lewy bodies and Alzheimer's disease, which are all caused by neurodegeneration (Piggott et al., 1999; Martorana and Koch, 2014). Understanding the molecular basis of neurodegenerative mechanisms is a complex and rapidly developing focus of research in neuropathology. It is currently believed that the major mechanism of neurodegeneration is the formation of the amyloid plaque which causes progressive loss of neural function and ultimately neuron death (Ross and Poirier, 2004; Chiti and Dobson, 2017). An alternative mechanism meanwhile, is the chemical damage of cellular membranes and proteins by reactive radical species, which causes leakage of the membrane and dysfunction of proteins, with subsequent loss of cell function and neuron death. The main sources of reactive oxygen species (ROS) in the CNS are the electron transfer chain, degradation of biogenic amines by monoamine oxidases (MAO), dopamine and noradrenaline autoxidation, and inflammatory processes (Graham, 1978; Pavlin et al., 2016; Herrera et al., 2017). Since the past few years, we have studied MAO-catalyzed decomposition of several monoamines, along with irreversible MAO inhibition, with the use of molecular simulations (Borštnar et al., 2011; Vianello et al., 2012; Pavlin et al., 2013; Repič et al., 2014b; Mavri et al., 2016; Poberžnik et al., 2016; Oanca et al., 2017a,c). In the present study, we attempted to further our understanding of monoamine decomposition reactions by addressing the autoxidation mechanism of dopamine degradation, which occurs in an aqueous solution rather than in an enzyme environment.

### Experimental Facts: Dopaminergic Synapse

In catecholaminergic neurons dopamine is synthesized in the cytosol near the synaptic vesicle (Cartier et al., 2010) and readily transported into them by vesicular monoamine transporter 2 (VMAT-2) (Chaudhry et al., 2008). Inside the synaptic vesicle, dopamine stability is aided by its storage in acidic environment, with a pH of ~5.6, as against the neutral cytosolic pH of ~7.1 and the extracellular pH of ~7.4 (Raley-Susman et al., 1991; Schwiening and Boron, 1994; Vincent et al., 1999; Mani and Ryan, 2009). The major pathways of dopaminergic signaling in CNS include classical synaptic signaling (Nikolaus et al., 2007), and extrasynaptic volume transmission, effective in the distance range of a few μm, signaling to many neighboring as well as distant synapses and glial cells (Fuxe et al., 2010, 2012; Uchigashima et al., 2016).

### Experimental Facts: Spontaneous Dopamine Oxidation

Dopamine autoxidation occurs in an aqueous solution without enzymes such as MAO, catechol-O-methyltransferases (COMT) or diamine oxidases (DAO). Dopamine first reacts with oxygen, giving rise to the quinone form and superoxide anion, the latter further decomposing to various ROS. The quinone form is capable of intramolecular Michael addition (cyclization), yielding aminochrome, which at physiological conditions represents the rate-determining step. The aminochrome can further polymerize into dark neuromelanin polymer (Baez et al., 1994). Dopamine autoxidation has been the subject of several kinetic studies (Tse et al., 1976; Young and Babbitt, 1983; García-Moreno et al., 1991; Herlinger et al., 1995; Lloyd, 1995; Hermida-Ameijeiras et al., 2004; Salomäki et al., 2018). Garcia-Moreno et al. have shown that cyclisation reaction is very fast at pH higher than 7.0, while at pH lower than 4.5 the dopamine autoxidation intermediates starts to accumulate, suggesting that cyclisation is the rate-limiting step of dopamine autoxidation at acidic pH (García-Moreno et al., 1991). Herlinger et al. measured the kinetics of dopamine autoxidation in the pH range of 7–9, inferring a strong pH-dependence and demonstrating that the rate-limiting step does not involve radical species (Herlinger et al., 1995). Lloyd also reported very strong pH-dependent kinetics where the rate constant exponentially depends on pH, and further showed that heavy metal ions such as manganese additionally catalyze the reaction (Lloyd, 1995). Very recently Salomäki et al. have confirmed that at pH below 5, the intramolecular Michael addition that gives rise to the cyclic aminochrome represents the rate-limiting step (Salomäki et al., 2018).

## Materials and Methods

The reaction mechanism of dopamine autoxidation was studied using quantum chemical calculations. Initial structures of dopamine, complexed with various species, were built using the Molden v5.8 software package (Schaftenaar and Noordik, 2000). The initial geometries of the structures were optimized at the M06-2X/6-31+G(d,p) level which is a good compromise between the computational costs and the reliability of results. The effects of solvation were considered by applying the solvent reaction field of Tomasi and coworkers (Miertuš et al., 1981). In the aforementioned solvation model, the solute cavity is composed of interlocking spheres and the solvent is described as a dielectric continuum. To represent the aqueous solution, a dielectric constant of 78.3 which corresponds to the experimental dielectric constant of bulk water, was applied and geometries of all structures were re-optimized by including the solvent reaction field (Schutz and Warshel, 2001). By inclusion of solvent reaction field, the Born–Oppenheimer surface obtains meaning of free energy surface and from stationary points one can draw conclusions about thermodynamics and kinetics. The same procedure was applied for the products' minima. Transition state search started from the manually-set initial geometry. For all stationary points, a vibrational analysis was performed in the harmonic approximation. Calculated frequencies allowed for zero-point correction of reaction and activation free energies. Reactants' and product' minima were proven to be real minima by having all real frequencies, while the transition states should have one imaginary frequency and the corresponding eigenvector of the latter should describe the reactive motion. Calculations were performed with the Gaussian 09 software package (Frisch et al., 2016). Such quantum chemical methods with proper inclusion of solvent effects, represent an established tool for studying binding and reactivity (Pisliakov et al., 2009; Eizaguirre et al., 2011; Rosta and Warshel, 2012; Sharir-Ivry et al., 2015; Khan et al., 2018).

Free energy differences, associated with proton transfer from the ionizable group with a certain pK_a_ value to bulk water with a certain pH value, were calculated using the following equation (Equation 1)

(1) $$\Delta G^{\neq }=k_{B}T\ln\left(10\right)\left(pKa-pH\right)$$

We considered a few possible reaction mechanisms and the results are described below.

## Results

### Dopamine O-Quinone Does not React With Two Water Molecules

**Figure 1:**
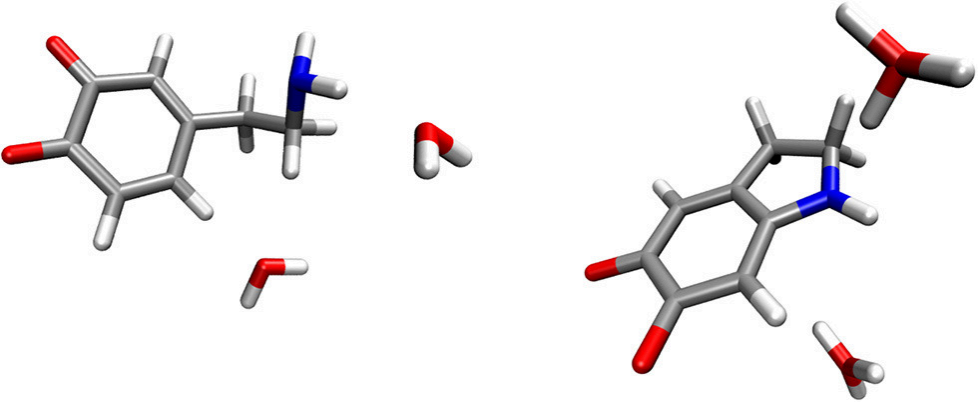
Optimized geometries (energy minima) of the reactants **(left)** and products **(right)** complexes. Reactants consist of a complex between dopamine o-quinone and two water molecules. Products consist of cyclized double charged dopamine o-quinone and two hydronium ions (H_3_O^+^). Carbon atoms are colored gray, oxygen atoms red, hydrogen atoms white and nitrogen atoms blue.

The reactants complex consisted of a dopamine o-quinone molecule and two water molecules. Two water molecules were placed so that they would act as proton acceptors, one in the hydrogen bond with the amino group (-NH_2_), and the other in the weak hydrogen bond involving the scissile C-H hydrogen atom. To prevent rupture of the hydrogen bonds in the geometry optimization process, both C-O and N-O distances were constrained to 3 Å for the reactants complex. For the products complex, consisting of one aminochrome molecule and two hydronium ions, all six O-H distances in both hydronium ions were constrained to 0.963 Å, as suggested by Kollman et al. (Kollman and Bender, 1973). All other degrees of freedom were fully optimized. The resulting optimized geometries for the reactants and products complexes are shown in Figure 1. A comparison of energies corresponding to the energy minima reveals that the reaction is strongly endergonic with the reaction free energy of 148.51 kcal mol^−1^, clearly proving that the reaction does not proceed via this mechanism. Therefore, we did not advance with transition state search and zero-point correction for this mechanism.

### Dopamine O-Quinone Probably Does Not React Simultaneously With Two Hydroxide Ions

The reactants complex, consisting of a dopamine o-quinone molecule and two hydroxide ions, was built so that the oxygen atoms of both hydroxide ions were acting as proton acceptors, one in the hydrogen bond with the amino group (-NH_2_), and the other in the weak hydrogen bond involving scissile C-H hydrogen atom. The products complex involved one aminochrome molecule and two water molecules. A comparison of energies between the minima shows the reaction is strongly exergonic with the reaction free energy of −62.70 kcal mol^−1^. However, this reaction mechanism is not plausible since the formation of two hydroxide ions at pH = 7.4 requires $2\times k_{B}T\ln\left(10\right)\left(15.7-7.4\right)=22.6\;kcal\;mol^{-1}$in terms of free energy, taking into account the experimental pK_a_ = 15.7 value for water molecule (Bronsted, 1928). Please note that the intrinsic barrier for the reaction with two hydroxide ions plus free energy of deprotonation of the dopamine o-quinone amino group should be added to this value. Dopamine o-quinone has a pK_a_ value of 9.58 (Young and Babbitt, 1983), therefore protonated amino group (-${\text{NH}}_{3}^{+}$) deprotonation cost in terms of free energy equals to $k_{B}T\;\ln\left(10\right)\left(9.58-7.4\right)=2.96\;kcal\;mol^{-1}$.The numerical procedure to locate the transition state and calculate the activation-free energy failed. From this it can be concluded that the mechanism utilizing a simultaneous attack by two hydroxide ions is probably not plausible, due to a too high activation-free energy.

### Dopamine O-Quinone Reacts With One Hydroxide Ion

**Figure 2:**
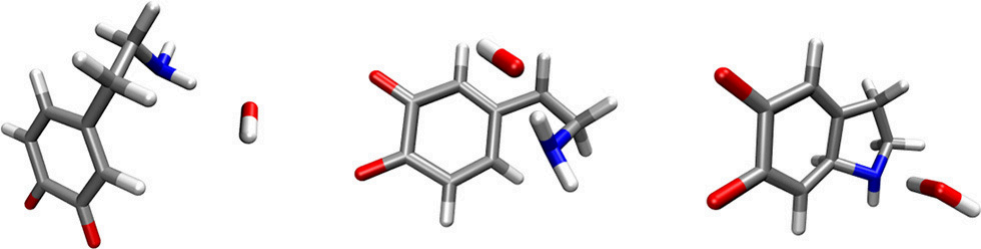
Optimized geometries (energy minima) of the reactants **(left)**, transition state **(middle)** and products **(right)** complexes. Reactants consist of a complex between dopamine o-quinone and a hydroxide ion (OH^−^). Products consist of cyclized single charged dopamine o-quinone and a water molecule. Carbon atoms are colored gray, oxygen atoms red, hydrogen atoms white and nitrogen atoms blue.

A hydroxide ion can form a hydrogen bond with, and abstracts a proton from either the amino group or the scissile C-H group. Both possibilities were considered. In the scenario where the hydroxide ion attacked the C-H group, the reaction free energy was 9.67 kcal mol^−1^, suggesting that this is not the preferred reaction channel. Moreover, cyclization did not occur. In the scenario where the hydroxide ion attacked the neutral amino group, the reaction-free energy was −27.79 kcal mol^−1^. The corresponding activation free energy was 6.78 kcal mol^−1^. The optimized geometries of the reactants, transition state and products complexes for this reaction step are shown in Figure 2.

**Figure 3:**
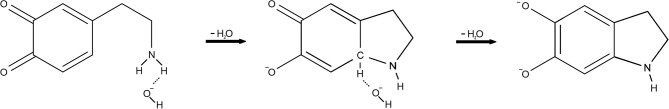
The reaction mechanism of dopamine autoxidation. Our calculations give evidence that at physiologic pH the rate-limiting step is the formation of a hydroxide ion (OH^−^) from a water molecule, which then attacks the amino group (-NH_2_) of dopamine o-quinone This is preceded by amino group deprotonation, since protonated amino group (-${\text{NH}}_{3}^{+}$) does not enter the intramolecular Michael addition. After the above-noted step, abstraction of the C-H proton by an additional hydroxide ion (OH^−^) follows.

In order to calculate the complete activation free energy for this rate-limiting step, which would be comparable to the experimental value, it is necessary to add the following to the intrinsic activation-free energy: the free energy of hydroxide ion formation $k_{B}T\;\ln\left(10\right)\left(15.7-7.4\right)=11.3\;kcal\;mol^{-1}$, and the free energy of deprotonation of the dopamine o-quinone amino group $k_{B}T\;ln\left(10\right)\left(9.58-7.4\right)=2.96\;kcal\;mol^{-1}$. Please note that the protonated amino group (-${\text{NH}}_{3}^{+}$) does not enter the intramolecular Michael addition, as is also the case for MAO catalyzed decomposition of biogenic amines (Oanca et al., 2017b). As such, the calculated activation-free energy for this rate-limiting step equals 21.0 kcal mol^−1^. After the above-noted step, another step follows-abstraction of the C-H proton by an additional hydroxide ion. For this latter step, the transition state was located with the classical barrier of 1.54 kcal mol^−1^. After vibrational energy correction for both the reactants and the transition state, this reaction step proved to be barrier-less. Formation of the hydroxide ion (11.3 kcal mol^−1^) is therefore the only constituent of the energy barrier for this second step, and consequently does not control the overall reaction rate. The rate-limiting reaction mechanism is depicted in Figure 3.

### Dopamine O-Quinone Autoxidation Is Strongly pH-Dependent

Suggested mechanism of dopamine reaction with one hydroxide ion allows us to analytically express the activation-free energy as a function of the pH value of the solution using the following equation (Equation 2):

(2) $$\begin{array}{l} \Delta G^{\neq }=6.78\;kcal\;mol^{-1}+k_{B}T\ln\left(10\right)\left(15.7-pH\right)\\ \;+k_{B}T\ln\left(10\right)\left(9.58-pH\right) \end{array}$$

Note that at acidic pH, the free energy cost for hydroxide ion and neutral amino group formation is much higher than at neutral pH. Inserting this expression into the transition state equation which links activation energy ΔG^≠^ with the reaction rate constant k_rate_.

(3) $$k_{rate}=\frac{k_{B}T}{h}e^{-(\frac{\Delta G^{\neq }}{k_{B}T})}$$

yields the pH-dependence:

(4) $$k_{rate}\left(pH\right)=\frac{k_{B}T}{h}e^{-(\frac{6.78\;kcal\;mol^{-1}+k_{B}T\ln\left(10\right)\left(15.7-pH\right)+k_{B}T\ln\left(10\right)\left(9.58-pH\right)}{k_{B}T})}$$

from which we can derive the following (Equation 5):

(5) $$k_{rate}\left(pH\right)=10^{-(c-2pH)}$$

The above derived functional equation yields results that are fully consistent with the dependence of the dopamine autoxidation rate constant upon pH-described by Lloyd (1995).

**Figure 4:**
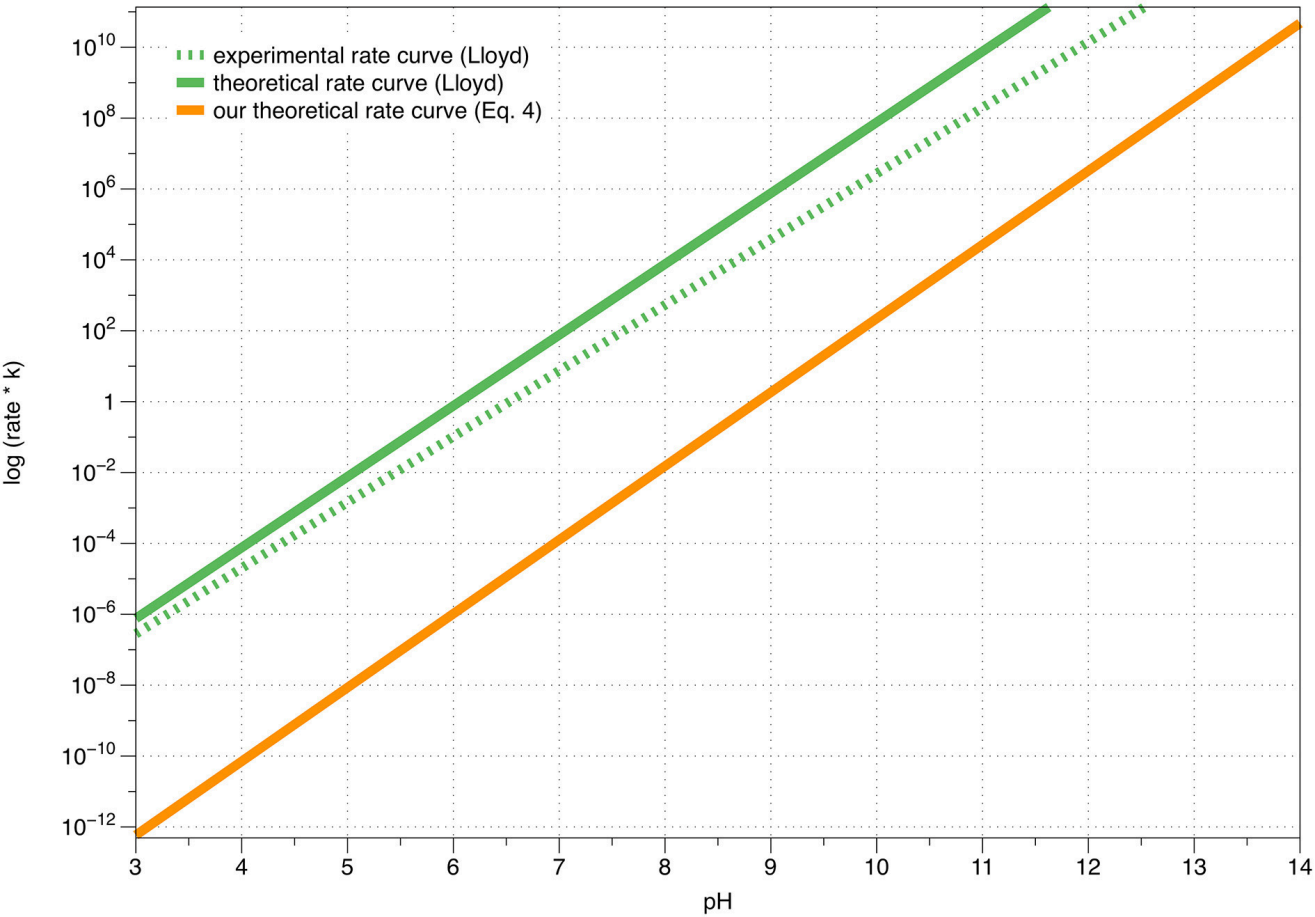
A graphical comparison between Lloyd's experimental, Lloyd's theoretical and our own calculated theoretical (Equation 4) rate constants of dopamine autoxidation in aqueous solution, as a function of the pH value.

In Table 1, a few rate constant values are provided, calculated using Equation 4. The corresponding pH-dependence of the rate constant is graphically shown in Figure 4. Note that the function is shifted horizontally relative to the experimental k_rate_ described by Tse et al. and Lloyd, who performed the experiments in the presence of magnesium ions that might change the kinetic data to certain extent, while the shape is practically identical to the function shown by Lloyd (Tse et al., 1976; Lloyd, 1995).

**Table 1:** A few rate constants and the corresponding reaction half-times for dopamine autoxidation in an aqueous solution as a function of the pH value, calculated using Equation 4.

**pH**	**k_**rate**_(s^**−1**^)**	**t_**1/2**_**
5.6	0.000000591	13.5 days
7.1	0.000586	19.7 min
7.4	0.00233	4.95 min
8.0	0.0368	18.8 s
9.58	52.8	0.0131 s

## Discussion

The results of the presented quantum chemical calculations show that the rate-limiting step of dopamine autoxidation is controlled by a sum of three free energy values: (1) formation of a hydroxide ion in water; (2) dopamine o-quinone charged amino group (-${\text{NH}}_{3}^{+}$) deprotonation; and (3) the intrinsic barrier for the reaction. The latter value is pH-independent, while the entire process is strongly pH-dependent. At a pH lower than 5.6, dopamine has a half-life on a time scale of days, while at a pH of 7.4, the entirety of dopamine reserves would disappear in only a few minutes. This emphasizes the role of the acidic interior of synaptic vesicles in dopamine stability and metabolism.

Autoxidation of dopamine has been investigated since the past few decades (Young and Babbitt, 1983; Herlinger et al., 1995; Lloyd, 1995; Hermida-Ameijeiras et al., 2004; Salomäki et al., 2018). Dopamine o-quinone cyclization at physiological pH values is very fast, with a rate constant of 0.147 s^−1^ and a half-life of 4.7 s, making the reaction mechanism almost impossible to investigate experimentally (Tse et al., 1976). To overcome this limitation, we used quantum chemical computation methods to elucidate the exact reaction mechanism, further utilizing it to explain the mechanism of strong pH-dependence. We found that at physiological pH, the rate-limiting step is the formation of a hydroxide ion. Our analytical expression for the pH-dependence of the rate constant (Equation 4) is fully consistent with the experimental pH-dependence shown by Lloyd (1995) and Salomäki et al. (2018), affirming the validity of our results.

The calculated activation-free energy for the cyclization reaction equals 21.0 kcal mol^−1^. This is reasonably close to the experimental barrier of 18.5 kcal mol^−1^ (Tse et al., 1976) at pH value of 7.4, considered that experimental determination of pK_a_ values of reaction intermediates and ionizable protein groups is not easy (Mildvan et al., 2002; Bezençon et al., 2014). Calculations concerning pK_a_ values are extremely demanding, typically involve errors of at least one pK_a_ unit, and thus represent a very strict test of the applied computational methodology (Warshel, 1981; Warshel et al., 2006; Borštnar et al., 2012; Repič et al., 2014a). An important source of error is also the calculation of hydration-free energy of the hydroxide ion that enters the reaction, as its negative charge gradually redistributes over the entire reactive complex throughout the course of the reaction. For ionic species, the hydration-free energy error calculated using a solvent reaction field is about 3 kcal mol^−1^ (Kelly et al., 2006). This accounts for the discrepancy between the experimentally-derived and calculated activation-free energy and rate constants.

Gu et al. demonstrated that in dopaminergic neurons, dopamine is released by a transient reversible fusion mechanism known as “kiss and run” (Gu et al., 2015). These characteristics could be understood as neuron adaptations for handling with neurotoxic dopamine by ensuring faster synaptic vesicle reacidification. Another strategy could be a physical and functional coupling of the enzymes for dopamine synthesis and VMAT-2, which immediately after synthesis transports and stores dopamine into acidic synaptic vesicles (Cartier et al., 2010). A third plausible mechanism to combat dopamine toxicity could be the physical and functional coupling of dopamine transporter (DAT) and VMAT-2 by scaffold proteins (Egaña et al., 2009), resulting in re-uptaken dopamine being immediately transported into the acidic vesicle interior.

In the case of classic synaptic transmission from presynaptic to the postsynaptic neuron, the synaptic gap is only 20–30 nm wide, which makes it very sensitive to pH change resulting from the release of protons from synaptic vesicles and the transient incorporation of vesicular H^+^-ATPase into the presynaptic membrane (Sinning and Hübner, 2013). Such brief but strong acidification of the synaptic gap and the resulting stabilization of dopamine could be essential for the classical synaptic dopamine transmission in the *corpus striatum*, where dopamine uptake by DAT is the most important determinant of dopamine signaling (Rice and Cragg, 2008). In the midbrain however, where volume transmission at a distance scale of μm takes place, dopamine rapidly diffuses to extrasynaptic regions where the pH is above 7 (Cragg et al., 2001). In such setting, the major factors influencing the time course of dopamine signaling are the diffusion rate and the pH-dependent rate of dopamine autoxidation.

Parkinson's disease is a neurodegenerative disease localized to the nigrostriatal pathway, where the major pathophysiological process is a degeneration of dopaminergic neurons. Despite intensive research into Parkinson's disease pathophysiology, the initial trigger of neuron degeneration is still unknown. It is generally accepted that α-synuclein aggregation, mitochondrial dysfunction, neuroinflammation, dysfunction of protein degradation and oxidative stress are involved in neurodegeneration. In the last decades, it has been shown that aminochrome, the product of dopamine autoxidation, can induce most of these changes and that the rate of some of them is pH dependent (García-Moreno et al., 1991; Paris et al., 2010; Segura-Aguilar et al., 2014; Herrera et al., 2017; Segura-Aguilar and Huenchuguala, 2018), suggesting that dopamine autoxidation could be one of the major pathophysiologic processes, and possibly even the major cause of Parkinson's disease. Mosharov et al. showed that elevated cytosolic concentrations of dopamine and its metabolites are cytotoxic, and noted that dopaminergic neurons in *substantia nigra* are especially susceptible to dopamine neurotoxicity compared to the more resistant dopaminergic neurons in the ventral tegmental area. They partially attributed this effect to higher cytosolic dopamine concentrations in neurons of *substantia nigra* (Mosharov et al., 2009), which can potentially explain the localized nature of Parkinson's disease.

The rate of dopamine autoxidation outside the synaptic vesicles is probably comparable to a MAO-B-catalyzed reaction (Pavlin et al., 2016). An indirect indication supporting this assumption is the fact that only a slight increase of extracellular dopamine concentration and subsequent excitation of postsynaptic dopamine receptors can be seen following an irreversible inhibition of MAO A or MAO B by phenelzine, selegiline or rasagiline (McKim et al., 1983; Kaseda et al., 1999; Albreht et al., 2018). This could explain the relatively modest effect of selegiline and rasagiline monotherapy in the treatment of Parkinson's disease (Dezsi and Vecsei, 2017), and also raises a concern regarding MAO B inhibition, which would as such increase dopamine autoxidation and subsequent aminochrome formation. Note that MAO B is located at the outer mitochondrial membrane, where are ROS scavenging enzymes are also colocated (Goldstein et al., 2017).

The amount of autoxidized dopamine and therewith associated ROS production is proportional to the level of cytosolic dopamine. Inhibition of DAT and VMAT-2 by amphetamines, leads to increased levels of dopamine in the synaptic gap, cytoplasm and extracellular space (Ciccarone, 2011). All three compartments have a pH of about 7, giving rise to rapid dopamine autoxidation. This could explain the increased susceptibility to development of Parkinson's disease in long-term amphetamine users (Lloyd et al., 2006; Curtin et al., 2015). Cytosolic dopamine levels are also dependent on the level of dopamine precursors and the activity of the catalytic enzymes that include phenylalanine hydroxylase (PAH) that is cofactor tetrahydrobiopterin (BH4) dependent. Thus, chronic CNS inflammation pathologies where IFNγ induces BH4 synthesis might lead to increased dopamine levels and increased incidence of neurodegeneration (Murr et al., 2014).

Since pH is physiologically very strictly regulated, its manipulation probably cannot be used to modulate dopamine autoxidation rate *in vivo*. Moreover, Pham et al. showed that the formation of insoluble α-synuclein oligomers is increased at acidic pH (Pham et al., 2009). It has also been suggested that at slightly acidic pH a toxic 6OH-dopamine could be formed during dopamine autoxidation (García-Moreno et al., 1991). Therefore, we believe that the best strategy to ameliorate dopamine autoxidation toxicity is to reduce or eliminate its toxic by-products such as ROS and aminochrome by modulation of activity of various antioxidants and the enzymes including glutathione-S-transferase M2 (GSTM2) and DT-diaphorase (Pavlin et al., 2016; Herrera et al., 2017).

Our study has a few limitations. First, the applied computational methods were limited to the quantum-chemical description of the reactive species, while solvent effects were included on the level of solvent reaction field. Explicit description of solvent, counterions and thermal fluctuations on the level of multiscale simulation is computationally demanding and would not significantly change the results (Warshel and Levitt, 1976). Second, we studied autoxidation of dopamine in aqueous solution, while the influence of other molecules or ions were not investigated. Third, the current study is limited to dopamine autoxidation and does not include pathways of dopamine synthesis and enzymatic decomposition. The latter is catalyzed by MAO B and represents additional source of reactive oxygen species (Pavlin et al., 2016).

## Conclusion

We have studied the reaction mechanism of dopamine autoxidation using quantum chemical methods, and shown that at the physiologic pH the rate-limiting step is the formation of a hydroxide ion. An analytical solution for describing the pH-dependence of the rate constant is derived. The proposed model is fully consistent with experimental data. A strongly pH-dependent rate constant supports the assumption that the rate-limiting step involves a heterolytic reaction and that a reaction mechanism involving free radicals is not plausible at physiological conditions. Therefore, for stability of dopamine stored in synaptic vesicles, an acidic interior is essential, since at neutral pH, dopamine would quickly almost completely disappear. We believe that dopamine autoxidation is one of the most important mechanisms in pathophysiology of Parkinson's disease, and the major factor for its localized nature on the nigrostriatal pathway.

## Author Contributions

NU, JM, and BG performed the calculations; NU, JM, BG, NV, and MŠ collected the literature and wrote the manuscript.

### Conflict of Interest Statement

The authors declare that the research was conducted in the absence of any commercial or financial relationships that could be construed as a potential conflict of interest.
